# Phase I study with ONCOS-102 for the treatment of solid tumors – an evaluation of clinical response and exploratory analyses of immune markers

**DOI:** 10.1186/s40425-016-0121-5

**Published:** 2016-03-15

**Authors:** Tuuli Ranki, Sari Pesonen, Akseli Hemminki, Kaarina Partanen, Kalevi Kairemo, Tuomo Alanko, Johan Lundin, Nina Linder, Riku Turkki, Ari Ristimäki, Elke Jäger, Julia Karbach, Claudia Wahle, Matti Kankainen, Charlotta Backman, Mikael von Euler, Elina Haavisto, Tiina Hakonen, Raita Heiskanen, Magnus Jaderberg, Juuso Juhila, Petri Priha, Laura Suoranta, Lotta Vassilev, Antti Vuolanto, Timo Joensuu

**Affiliations:** Oncos Therapeutics, Helsinki, Finland; Cancer Gene Therapy Group, Hartman Institute, University of Helsinki, Helsinki, Finland; Helsinki University Hospital Comprehensive Cancer Center, Helsinki, Finland; Docrates Cancer Center, Helsinki, Finland; The University of Texas MD Anderson Cancer Center, Houston, TX USA; Institute for Molecular Medicine Finland (FIMM), Helsinki, Finland; Pathology, Research Programs Unit and HUSLAB, University of Helsinki and Helsinki University Hospital, Helsinki, Finland; Hämatologie-Onkologie, Krankenhaus Nordwest, Frankfurt, Germany; Medix Biochemica, Helsinki, Finland; Merck Sharp & Dohme, Espoo, Finland

**Keywords:** Immunotherapy, *in situ* vaccine, Cytotoxic CD8+ T cell, Anti-tumor immunity, Intratumoral, Oncolytic adenovirus

## Abstract

**Background:**

We conducted a phase I study with a granulocyte macrophage colony stimulating factor (GMCSF)-expressing oncolytic adenovirus, ONCOS-102, in patients with solid tumors refractory to available treatments. The objectives of the study were to determine the optimal dose for further use and to assess the safety, tolerability and adverse event (AE) profile of ONCOS-102. Further, the response rate and overall survival were evaluated as well as preliminary evidence of disease control. As an exploratory endpoint, the effect of ONCOS 102 on biological correlates was examined.

**Methods:**

The study was conducted using a classic 3 + 3 dose escalation study design involving 12 patients. Patients were repeatedly treated intratumorally with ONCOS-102 plus daily low-dose oral cyclophosphamide (CPO). Tumor response was evaluated with diagnostic positron emission tomography (PET) and computed tomography (CT). Tumor biopsies were collected at baseline and after treatment initiation for analysis of immunological correlates. Peripheral blood mononuclear cells (PBMCs) were collected at baseline and during the study to assess antigen specificity of CD8+ T cells by interferon gamma (IFNγ) enzyme linked immunospot assay (ELISPOT).

**Results:**

No dose limiting toxicity (DLT) or maximum tolerated dose (MTD) was identified for ONCOS-102. Four out of ten (40 %) evaluable patients had disease control based on PET/CT scan at 3 months and median overall survival was 9.3 months. A short-term increase in systemic pro-inflammatory cytokines and a prominent infiltration of TILs to tumors was seen post-treatment in 11 out of 12 patients. Two patients showed marked infiltration of CD8+ T cells to tumors and concomitant systemic induction of tumor-specific CD8+ T cells. Interestingly, high expression levels of genes associated with activated T_H_1 cells and T_H_1 type immune profile were observed in the post-treatment biopsies of these two patients.

**Conclusions:**

ONCOS-102 is safe and well tolerated at the tested doses. All three examined doses may be used in further development. There was evidence of antitumor immunity and signals of clinical efficacy. Importantly, treatment resulted in infiltration of CD8+ T cells to tumors and up-regulation of PD-L1, highlighting the potential of ONCOS-102 as an immunosensitizing agent for combinatory therapies with checkpoint inhibitors.

**Trial registration:**

NCT01598129. Registered 19/04/2012

## Background

The concept of oncolytic viruses as cancer therapeutics has gained considerable attention over the last decade while expectations regarding the prospect of long lasting clinical responses with viral therapy are yet to be fulfilled. The first oncolytic virus entered the market recently when FDA approved T-VEC, a herpes simplex virus coding for GM-CSF, for the treatment of advanced melanoma [1]. With the recent excitement around new immunotherapeutic approaches, especially the concept of checkpoint molecule blockade, there has been a clear shift in the way viral cancer therapy is regarded from providing mainly oncolysis towards being an immunologic form of cancer treatment [2, 3].

The presence of infiltrating immune cells in the tumor is now recognized as an important prognostic factor associated with the clinical outcome of many cancer types [4, 5]. In addition, the localization within the tumor, as well as the type and functionality of the immune cell infiltrates, have a major influence on the host-tumor interactions [4–6]. However, with the recent advances in the development of checkpoint modulator molecules targeting the negative feedback mechanisms that suppress CD8+ T-cell effector functions, it has become evident that immune cell-poor cancers are not an optimal target group for this class of immunotherapy, unless coupled to an immune priming agent [7, 8].

Immune cell infiltration to tumor is a frequent consequence of treatment with oncolytic viruses, [9] making them potential immune primers. Adenoviruses are good immunotherapeutic agents due to their high immunogenicity. They can both prime and boost cellular and humoral immune responses, [10] which is why they are frequently used as vaccine platforms [11]. Importantly, adenoviruses cause cellular immunity with induction of CD8+ T-cells, key effector cells in cancer immunity [2, 3]. Adenoviruses cause immunogenic cancer cell lysis where upon tumor antigens previously hidden from the immune system or not presented in an immunogenic context are released into the immunogenic environment. This results in an induction of T-cell response against tumor-derived antigens, including unique patient specific neoantigens. Furthermore, repeated treatment provides an update of the antigen repertoire presented to the immune system. Although the immune response to virus is strong, a CD8+ T-cell response to tumor antigens is likely to occur as well [12]. T-cell response may be further enhanced by immune-stimulating transgenes expressed by the virus.

ONCOS-102 is a serotype 5 adenovirus that has a genetically modified fiber with a serotype 3 knob for enhanced gene delivery to cancer cells [13]. It is armed with GM-CSF, to enhance antitumor immunity. GM-CSF recruits antigen presenting cells (APC) and natural killer (NK) cells. In addition GM-CSF activates and matures APCs at the tumor site, thereby potentiating the ability of ONCOS-102 to induce cellular immunity against the tumor it replicates in [14, 15]. To ensure selective replication in cancer cells and patient safety, 24 bp has been deleted in the Rb binding site of *E1A* gene [16].

Up to date, clinical studies of other frequently used viral platforms, including Herpes simplex virus 1 (HSV1) and vaccinia virus, have failed to show reliable and convincing results on virus-induced CD8+ T-cell immunity against cancer, even when the vectors were armed with GM-CSF [17–20]. This despite these clinical trials being conducted in melanoma that is known to be one of the most immunogenic cancer types [21]. In this phase I study, we assessed safety, efficacy and immunological endpoints of local treatment with a replicating adenovirus ONCOS-102 in 12 patients with late stage solid cancer of various types including mesothelioma, ovarian cancer, soft tissue sarcoma, colorectal cancer, liver and lung cancer. All patients had received chemotherapy and were treatment refractory, 66 % had had radiotherapy and 50 % surgery. The median time from diagnosis to study entry was only 2.5 years.

## Results

### Safety

Treatments were well tolerated without any grade 4–5 adverse events (AEs) (Table 1). Most AEs were of Grade 1 or Grade 2. Flu-like symptoms and pyrexia were common AEs, with fever being reported in every patient. A total of 15 Grade 3 AEs were reported in 6 patients, and in 5 patients were considered treatment-related: pyrexia, increased alkaline phosphatase (ALP), increased aspartate aminotransferase (AST), proteinuria, hyponatremia, anaemia, fatigue, oedema peripheral, and dyspnoea. There was no indication of a relationship between dose of ONCOS-102 and the incidence and intensity of AEs. DLT or MTD was not reached in this study.

**Table 1:** Number of patients with related adverse events by CTCAE grading

Related adverse events	CTCAE grade			Any grade
MedDRA preferred term	1	2	3	
Pyrexia	12	9	2	12
Chills	9	6		10
Fatigue	10	5	1	10
Injection site pain	8	3		9
Decreased appetite	8	1		8
Feeling cold	7	2		8
Hyperhidrosis	5	4		8
Nausea	6			6
Anaemia	3	4	1	5
Pain	3	3		5
Vomiting	5	2		5
Headache	3	3		4
Blood alkaline phosphatase increased		1	2	3
Injection site haematoma	3			3
Night sweats	2	2		3
Abdominal distension	2	1		2
Dyspnoea	1	1	1	2
Pneumonia		2		2
Somnolence	2			2
Upper respiratory tract infection		2		2
Aspartate aminotransferase increased			1	1
C-reactive protein increased		1		1
Dyspepsia		1		1
Hepatic pain	1	1		1
Hypoalbuminaemia			1	1
Hyponatraemia			1	1
Myalgia	1	1		1
Oedema peripheral		1	1	1
Oral herpes		1		1
Proteinuria			1	1
Abdominal pain upper, Arthralgia, Back pain, Cough, Dizziness, Dyspnoea exertional, Eczema, Haematoma, Hypovolaemia, Injection site haemorrhage, Iron deficiency, Libido decreased, Lymphadenopathy, Malaise, Muscle strain, Muscular weakness, Oedema, Peripheral coldness, Polyuria, Post procedural haemorrhage, Pruritus, Urticaria	1			1

No grade 4–5 events were reported

### Systemic pro-inflammatory cytokines

**Fig. 1:**
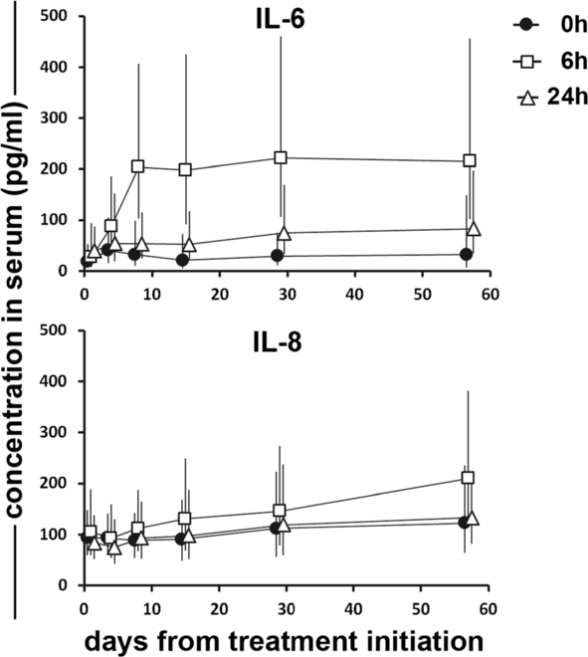
Intratumoral ONCOS-102 treatment triggered a short-term increase in the systemic levels of pro-inflammatory cytokines. Treatment with ONCOS-102 induced a short-term increase of systemic pro-inflammatory cytokines IL-6 and IL-8 in patients. The increase was most prominent 6 h after each treatment and decreased nearly to baseline-values by the 24-h time-point

A short-term increase in systemic pro-inflammatory cytokines was seen in all patients following treatment with ONCOS-102. Concentrations of interleukins 6 and 8 (IL-6 and IL-8) in serum peaked 6 h after intratumoral ONCOS-102 injections, indicating a rapid innate immune response towards treatment. Cytokines had decreased to values close to baseline by 24 h (Fig. 1). The fever that was seen concomitant with the peak in the systemic cytokine levels indicates that innate immunity was evoked by the treatment. Concentration of the anti-inflammatory cytokine interleukin 10 (IL-10) increased to some extent post-treatment, but the levels remained much lower when compared to the pro-inflammatory cytokines (data not shown). The level of tumor necrosis factor alpha (TNF-α) remained low or non-detectable in serum throughout the treatment period in all patients. The baseline level of systemic GM-CSF was undetectable or very low in all patients before the first treatment (range of 0–8 pg/mL). Two patients had post-treatment increase in systemic GM-CSF level after two injections. These patients showed an increase in the concentration of GM-CSF in serum 24 h after the second treatment (165 and 109 pg/mL). Since the timing of the peak of GM-CSF concentration is coincidental with the timing of the gene expression from the E3 replication cassette, it indicates productive viral replication and transgene expression in the tumor.

### Response according to RECIST 1.1 and PET response criteria

Of the 12 patients included, two patients passed away before the first clinical assessment leaving 10 patients evaluable for radiological response by CT-PET-imaging. Of these, 4 patients (40 %) had disease control (stable disease) at 3 months while all patients had progressed at 6 months according to RECIST 1.1 criteria (Table 2). Four patients had stable metabolic disease based on PET response at 3 months. Five patients had PET response data at 6 months, of which 1 patient had stable metabolic disease, and 4 patients had progressive metabolic disease. Median progression-free survival was 2.9 months (95 % confidence interval [CI]: 2.7 – 5.5). Median overall survival was 9.3 months (95 % CI: 3.6 – 12.7) in the per protocol population (*n* = 10) and 8.5 months (95 % CI: 3.0 – 12.7) in the intent-to-treat population (*n* = 12). Importantly, we saw a late decrease in metabolic activity in PET imaging of patient FI1-14 with malignant pleural mesothelioma. In this patient there was a 47 % decrease in total lesion glycolysis 6 weeks after the last study visit which occurred at 6 months. The patient had not received other treatments after the trial, suggesting that the decrease in metabolic activity was caused by ONCOS-102. We believe that the activated immune response may lead to the development of delayed yet long-lived memory response that can sustain clinical benefit beyond the period of treatment as is often seen with immune-oncology therapies [22]. This patient survived 18 months (542 days) from the treatment initiation and over 33 months (999 days) from diagnosis. Patient FI1-19 had an epithelial ovarian carcinoma and had progressive disease following seven different chemotherapy treatment lines while entering the trial and continued to progress during study participation. Interestingly, after the study she responded to chemotherapy and imaging of her tumor 22 months after the study initiation showed stable disease compared to baseline and she was alive in the last follow up 25 months after study initiation.

**Table 2:** Patient characteristics, prior treatments, response at 3 and 6 months and overall survival

Patient	WHO score	Cancer type	Previous treatments (other than chemotherapy)	Previous chemotherapy	Response at 3/6 months		Survival (days)
					RECIST 1.1	PET	
FI1-01	1	Ovarian carcinoma	Surgery, radiotherapy	Docetaxel + carboplatin, carboplatin, paclitaxel + carboplatin, paclitaxel, gemcitabine, PLD, etoposide, cisplatin, vinorelbine, topotecan, oxaliplatin, docetaxel, epirubicin, irinotecan, gemcitabine hydrochloride, tamoxifen	SD/PD	PMD/SMD	278
FI1-02	0	Metastatic colon carcinoma	Surgery	oxaliplatin, capecitabine, cetuximab + irinotecan + capecitabine	SD/PD	SMD/PMD	382
FI1-04	0	Adenocarcinoma in sigma	-	Oxaliplatin + capecitabine + bevacizumab, Capecitabine + bevacizumab, FolFiri + bevacizumab, xelox	PD/ n/a	PMD/ n/a	124
FI1-06	0	Hepatocellular carcinoma	-	sorafenib tosilate, ramucirumab	PD/ n/a	PMD/ n/a	109
FI1-08	1	Pulmonum adenocarcinoma	Radiotherapy	pemetrexed disodium + cisplatin, erlotinib hydrochloride, docetaxel	PD/ n/a	PMD/ n/a	155
FI1-09	1	Lung mesothelioma	-	pemetrexed + cisplatin, tramadol hydrochloride	PD/ n/a	SMD/ n/a	254
FI1-13	0	Rectal adenocarcinoma	Surgery	Oxaliplatin + capecitabine, bevacizumab, irinotecan + capecitabine, panitumumab	PD/ n/a	PMD/ n/a	290
FI1-14	1	Asbestos related pleural mesothelioma	Radiotherapy	Docetaxel, cisplatin + pemetrexed disodium	SD/PD	PMD/PMD	542
FI1-15	1	Serous endometrial cancer	Surgery, Radiotherapy	PLD, paclitaxel + carboplatin x 2, gemcitabine, topotecan, docetaxel	- ^a^	- ^a^	90
FI1-17	1	Soft tissue sarcoma	Surgery, Radiotherapy	Ifosfamide + doxorubicin, gemcitabine + docetaxel, letrozole, trabectedin, zoledronic acid, gemcitabine + docetaxel, pazopanib hydrochloride	PD/PD	PMD/PMD	330
FI1-18	1	Breast cancer	Surgery, Radiotherapy	Docetaxel *x*2, cyclophosphamide + epirubicin + fluorouracil x 2, tamoxifen citrate, letrozole, anastrozole *x*2, exemestane, bevacizumab, capecitabine, medroxyprogesterone acetate, vinorelbine tartrate, epirubicin, cisplatin + gemcitabine, cyclophosphamide + methotrexate sodium	- ^a^	- ^a^	63
FI1-19	0	Ovarian cancer	Surgery	Paclitaxel + carboplatin x 2, paclitaxel + cisplatin, docetaxel + cisplatin, topotecan, gemcitabine, etoposide	SD/PD	SMD/PMD	761^b^

*PLD* Pegylated liposomal doxorubicin hydrochloride, *FolFri* fluorouracil w/folinic acid/irinotecan, *SD* stable disease, *PD* progressive disease, *SMD* stable metabolic disease, *PMD* progressive metabolic disease

^a^Patient died before the imaging at 3 month time-point

^b^Patient alive on 16June2015, n/a = patient withdrawn from the trial

### Virus genomes in blood, urine and buccal swabs, and neutralizing antibodies in blood

**Fig. 2:**
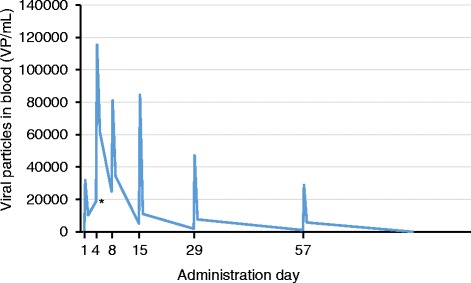
ONCOS-102 viral particles in blood. ONCOS-102 particles in blood were analyzed before each administration and 6 and 24 h after each administration. The number of viral genomes peaked at 6-h and lower values were detected 24-h after administration. A secondary peak in virus titer (*) suggests a productive virus replication at tumor site. Data is presented as median of all patients per time point

Adenoviruses are rapidly cleared from the bloodstream, [23] and therefore extended presence or increasing titers in serum suggest viral replication. We analysed the baseline and post-treatment serum samples for the presence of ONCOS-102 genomes by quantitative real-time PCR (qRT-PCR) to evaluate, whether signs of productive replication were evident after intratumoral treatment. All patients were negative for ONCOS-102 before the first treatment and had viral genomes present in the bloodstream 6 and 24 h post-treatment (Fig. 2). This was probably indicative of leakage from the injected tumor instead of productive replication, as the viral life-cycle lasts for 72 h. At later time-points 9/12 patients showed a viral titer that was higher than the 24-h titer after previous treatment, suggesting productive replication and cell lysis at the injected tumor.

To evaluate the possible virus shedding via urine and saliva, the presence of ONCOS-102 genomes was analysed in urine samples from all 12 patients and buccal swab samples from 9 patients treated in the two highest dose cohorts. All patients were negative for virus at baseline, but 4 and 4 patients had a quantifiable level of viral genomes present in urine and buccal swabs after treatment, respectively (data not shown). The urine and buccal swab samples were further used for viral cultures on A549 cells to verify, whether infective particles were present. A total of 3 patients were positive for infective virus 3 days after the first ONCOS-102 administration. One patient had infective viral particles present both in urine and buccal swab, while 2 patients showed infective virus only in buccal swabs. Importantly, positive samples were only collected after the first injection when 20 % of the dose was given intravenously. All subsequent samples were negative when the entire dose of ONCOS-102 was given intratumorally. This suggests that systemic administration leads to a more widespread biodistribution of virus compared to intratumoral administration.

8/12 patients were positive for neutralizing antibodies (NAb) at the baseline, with the NAb titer varying from <8 to 1024. Titers increased by day 29 in all evaluated patients. No correlation was seen between NAb titers and cytokine levels, immune cell infiltration or overall survival. Of note, patient FI1-19 had the highest baseline NAb titer among all patients but nevertheless showed the biggest post-treatment infiltration of CD8+ T cells in tumors and concomitant systemic induction of several tumor-specific CD8+ T cell populations, indicating that the presence of pre-existing NAbs did not affect the biological activity of the intratumoral injections of ONCOS-102.

### Treatment with ONCOS-102 resulted in prominent immune cell infiltration to tumors

**Fig. 3:**
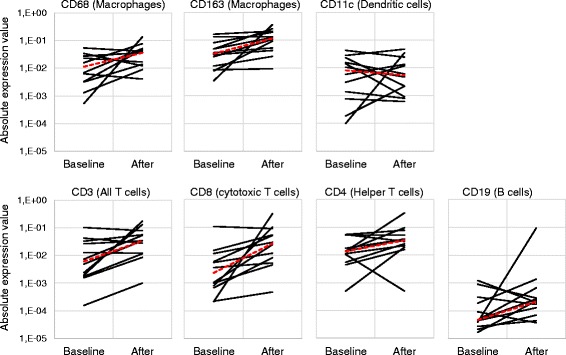
Intratumoral ONCOS-102 treatment induced an infiltration of immune cells to tumors. An absolute expression of indicated immune cell markers by immunohistochemistry in sequential tumor biopsies was quantified before and after local ONCOS-102 treatment. All 12 patients treated in the study are presented. Each solid line indicates an individual patient. Dotted line indicates median

**Fig. 4:**
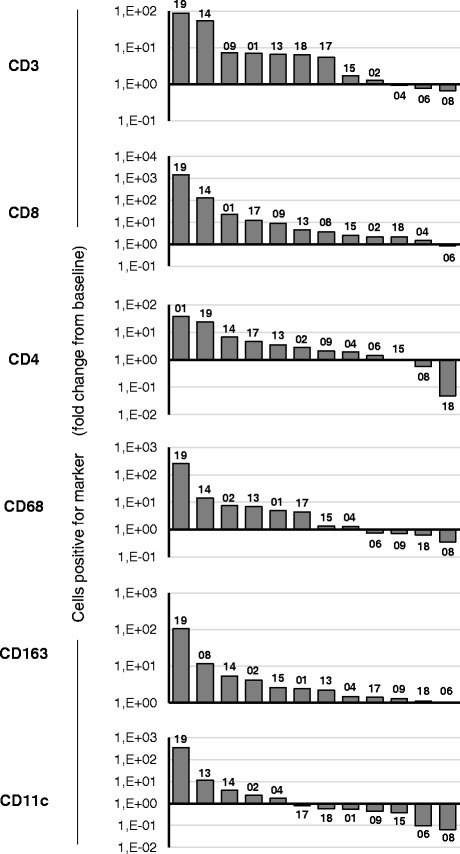
Increased number of immune cells were detected by immunohistochemistry in tumors after ONCOS-102 treatment. A waterfall plot of indicated CD markers for all patients treated in the trial. Results are presented as the biggest logarithmic fold-change from baseline either 1 month or 2 months after treatment. Patient code is depicted in each column

**Fig. 5:**
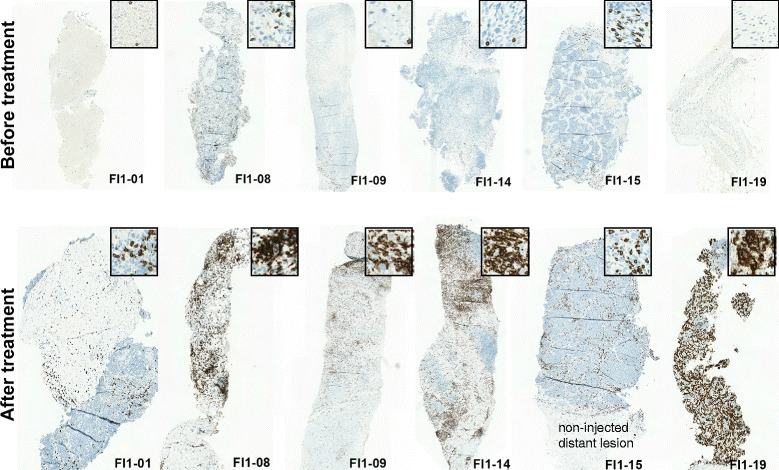
ONCOS-102 attracted CD8+ lymphocytes to tumors. A prominent infiltration of CD8+ T-cells was seen after treatment (lower row) in tumors showing very little CD8+ T-cells before treatment (upper row). Of note, patient FI1-15 showed infiltration of CD8+ immune cells to a non-injected distant tumor

**Fig. 6:**
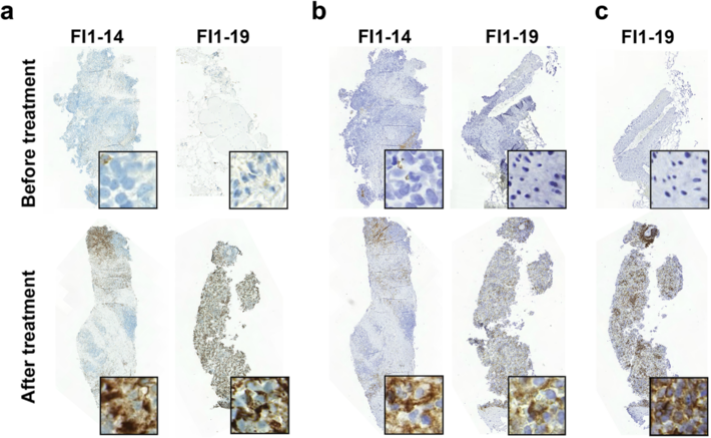
ONCOS-102 attracted macrophages and B cells to tumors**.** Infiltration of CD68+ (**a**), CD11c + (**b**) and CD19+ (**c**) immune cells in patients FI1-14 and FI1-19 was seen after treatment with ONCOS-102

**Fig. 7:**
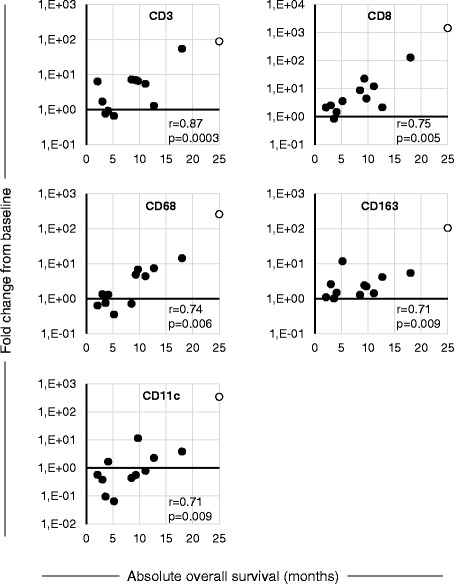
Increase in tumor-infiltrating immune cells following ONCOS-102 treatment is associated with increased survival. The fold change of total T cells (CD3+), CD8+ cells, CD68+ cells, CD163+ cells, and CD11c + cells correlated with overall survival in patients treated with ONCOS-102. Correlation between the post-treatment increase in different sub-populations of TILs and overall survival was assessed by Spearman’s rank correlation analysis. Overall survival is depicted as months, open label is patient FI1-19 who is still alive

Since the presence of TILs has been recognized as a marker of anti-tumor immune response across a wide range of tumors [5, 24–27] and a positive correlation has been linked to high TIL counts at pre-treatment samples and good prognosis, [28] we set out to determine whether treatment with ONCOS-102 induces immune cell infiltration to tumors. Immunohistochemical staining revealed that tumor samples taken at baseline before treatment had variable numbers of CD3+ cells, CD8+ cells, CD4+ cells, CD68+ cells, CD163+ cells, and CD11c + cells (Fig. 3). Generally very low numbers of CD19+ B cells were detected in tumors before and after ONCOS-102 treatment (Fig. 3). Following treatment with ONCOS-102, T cell marker CD3 showed an increased expression levels in post-treatment biopsies with the median fold-change from baseline being 5.9 in comparison to same tumors prior to treatment. ONCOS-102 treatment primarily attracted CD8+ T-cells in tumors with the median fold-change of 4.0 from baseline while the median fold-change for CD4+ T-cells was 2.5. Altogether, 11/12 patients showed a post-treatment increase in tumor infiltrating CD8+ cells compared to baseline (Figs. 4 and 5). The highest fold-increase was observed in the samples of patients FI1-14 and FI1-19 (131 fold and 1451 fold increase) (Figs. 4 and 5). Interestingly, patient FI1-15 also showed a clear infiltration of CD8+ cells in a non-injected tumor (Fig. 5), which is a possible indication of a systemic tumor-specific immunity evoked by the treatment, as the virus was given locally. Further, patients FI1-14 and FI1-19 also showed a clear increase in the number of CD68+ and CD11c + cells after treatment, suggesting infiltration of other immune cells, most likely macrophages and dendritic cells (Fig. 6a and b). TIL increase post-treatment was associated with prolonged survival. A statistically significant positive correlation between survival and infiltration of CD3+, CD8+, CD68+, CD163+, and CD11c + cells was seen (Fig. 7). Similarly, absolute numbers of macrophages (CD68+, CD163+) and T cells (CD3+, CD8+, CD4+) in post-treatment tumors positively correlated with OS (data not shown). Interestingly, the absolute expression level of CD68 (macrophage marker) at baseline negatively correlated with overall survival (*p* = 0.04, correlation coefficient (r) = −0.59) while baseline levels of other immune cell markers showed no correlation with OS. We suggest that tumor associated macrophages (CD68+ TAMs) present in baseline biopsies were tumorigenic and supported disease progression as has been suggested in the literature [29–31]. On the other hand, positive correlation between post-treatment TILs and OS suggest that ONCOS-102 was able to modulate local immunological microenvironment at tumors and recruit activated immune cells that had cytotoxic properties and were capable of slowing down the disease progression. Patient FI1-19 also showed a remarkable increase in the number of CD19+ cells, suggesting a prominent infiltration of B-cells to tumors (Fig. 6c). Tumor infiltrating B-cells have been suggested to enhance T-cell responses by serving as antigen presenting cells (APCs), and the co-localization of B-cells with CD8+ TILs has been shown to be associated with markedly increased survival compared with CD8+ TILs alone [32–34]. These results clearly demonstrate the capacity of intratumoral treatment with ONCOS-102 to evoke cellular immune responses in tumors that do not have immune cell infiltration before treatment.

### Tumor-specific cellular immune response

**Fig. 8:**
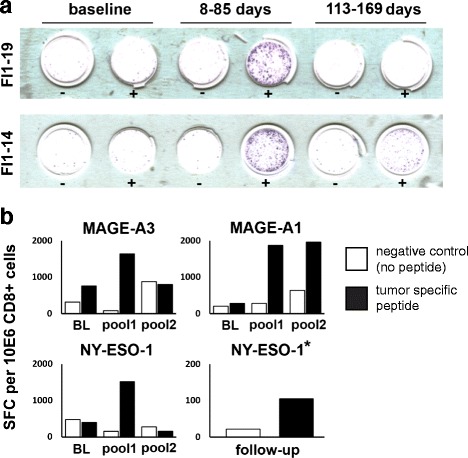
Intratumoral ONCOS-102 treatment induced systemic tumor-specific CD8+ T cell responses in chemotherapy refractory cancer patients. Systemic, tumor-specific CD8+ cellular response depicted in IFNγ ELISPOT. **a** Anti-MAGE-A3 ELISPOT for CD8+ cells in patients FI1-14 (lower row) and anti-mesothelin ELISPOT for CD8+ T cells for patient FI1 19 (upper row). **b** Numerical values for anti-MAGE-A3, anti-MAGE-A1 and anti-NY-ESO-1 (p157-165) ELISPOT for patient FI1-19. BL = baseline, pool1 = days 8–85 after treatment initiation, pool 2 = days 113–169 after treatment initiation. * = numerical values for anti-NY-ESO-1 (p91-110) ELISPOT at follow-up 17 months after the last ONCOS-102

Due to the general relative paucity of tumor antigen-specific T-cells as opposed to the antiviral T-cells, [35] we wanted to determine whether treatment with ONCOS-102 is able to induce tumor-specific CD8+ T-cell response. IFN-γ ELISPOT was performed from pre- and post-treatment PBMCs to determine specificity of CD8+ T-cells for cancer-testis (CT) antigens NY-ESO-1, MAGE-A1, and MAGE-A3. The differentiation antigen mesothelin was also analysed for patient FI1-19 diagnosed with ovarian cancer. Three patients showed either no IFN-gamma response (FI1-15) or only modest IFN-gamma response (FI1-02, FI1-08) to positive control and therefore could not be reliably assessed for tumor specific CD8+ T cell responses. Thus, 9 out of 12 patients were evaluable for tumor specific antigen responses, as their baseline and after treatment CD8+ T cells showed a clear IFN-gamma response upon stimulation with a CEF control peptide pool. All baseline samples were negative for tumor antigen specific CD8+ T-cells. Two patients had a clear induction of tumor recognizing CD8+ T cells as a result of the treatment. Pleural mesothelioma patient FI1-14 showed a prominent post-treatment induction of MAGE-A3-specific CD8+ T-cells in the early pool of CD8+ cells collected 8–85 days after treatment initiation, and MAGE-A3-recognizing CD8+ T cells were still present in the late pool of CD8+ T cells collected between days 113 and 169 (Fig. 8a). Ovarian cancer patient FI1-19 showed CD8+ T-cell responses against all three CT antigens (Fig. 8b) as well as mesothelin (Fig. 8a) in the early post-treatment pool of CD8+ cells and against MAGE-A1 in the late pool (Fig. 8b). Importantly, CD8+ T-cells specific for NY-ESO-1 were still present during follow up period in the blood sample collected 17 months after the last virus injection. These results suggest that intratumoral treatment with ONCOS-102 elicits a long-term, systemic *de novo* tumor specific immunity despite the presence of highly immunogenic viral antigens.

### T_H_1-type gene expression profile

Previously, the presence of a broad gene-signature of inflammation at the tumor was shown to be predictive for good prognosis in colon cancer patients [6, 36]. More specifically, this T_H_1-type gene-signature indicates innate immune activation, T-cell recruitment and expression of effector molecules as well as expression of immune regulatory factors in the tumor. We hypothesized that treatment with ONCOS-102 can induce a beneficial inflammatory environment within the tumor by inducing the expression of genes related to T_H_1-type gene signature. Gene expression profiling by microarray analysis revealed an increase in the expression of genes related to T_H_1-type signature, specifically markedly elevated expression levels of genes encoding cytotoxic factors perforin, granzyme B and granulysin post-treatment (Table 3), suggesting that the treatment-induced TILs displayed effector functionality. Further, elevated expression levels of genes encoding T_H_1 associated factors such as interferon gamma (IFNγ) and interferon regulatory factor 1 (IRF1), and T_H_1 associated chemokines (CCL2, RANTES, CX3CL1, CXCL9 and CXCL10) were seen post-treatment as well.

**Table 3:** Expression of genes related to a TH1 type gene signature before and after treatment in selected patients

	FI1-09		FI1-14		FI1-19	
Gene	baseline	after	baseline	after^a^	baseline	after
Granzyme B	7.3	8.5	7.2	10.1	na	9.9
Granulysin	8.3	7.2	7.1	9.0	na	8.9
Perforin	7.3	8.1	7.1	9.6	na	9.7
IFNγ	6.8	7.7	6.9	8.6	na	7.6
IRF1	9.3	10.7	10.6	11.9	na	11.9
RANTES	8.4	11.6	9.3	12.9	na	12.3
CXCL9	7.5	10.8	9.3	12.4	na	10.5
CXCL10	8.7	10.5	10.2	11.8	na	10.3

Baseline = before treatment, after = 2 months after the treatment initiation

^a^= 1 month after the treatment initiation

*IFN*γ interferon gamma, *IRF1* interferon regulatory factor 1

### Treatment with ONCOS-102 induces PD-L1 expression in the tumor

**Fig. 9:**
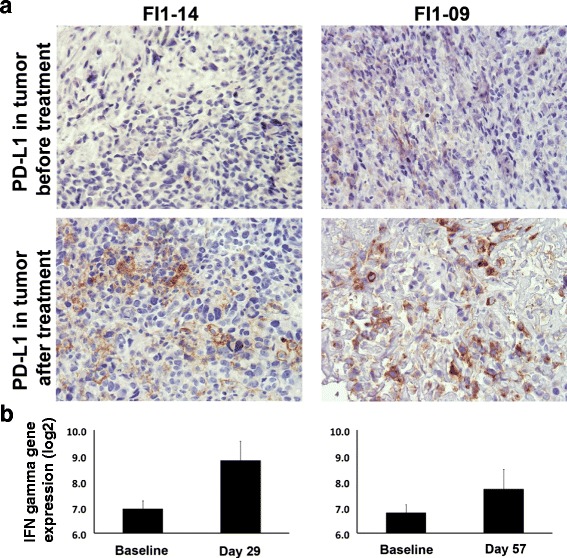
ONCOS-102 treatment induced up-regulation of PD-L1 in tumors. The increase in PD-L1 expression (**a**) coincided with an infiltration of CD8+ cells (Fig. 6) and an increase in gene expression of IFN-γ in tumors in mesothelioma patients FI1-14 and FI1-09 (**b**), suggesting induction of dynamic adaptive changes in response to T-cell-derived IFNγ

Therapies targeting the programmed death 1 (PD-1) receptor have shown great promise in cancer treatment, resulting in durable responses in various cancer types [37–42]. Recently it has been suggested that the presence of PD-1 expressing CD8+ T-cells at the invasive tumor margin and inside the programmed death 1 ligand (PD-L1) expressing tumor is a prerequisite for successful anti-PD-1 therapy [7]. Furthermore, an increased PD-L1 expression in tumor cells has been suggested to reflect the presence of active anti-tumor immune response [43]. Therefore, we wanted to analyse whether the treatment with ONCOS-102 has an impact on the PD-L1 expression status of the treated tumors. Indeed, we saw a clear post-treatment induction of PD-L1 expression in the tumors of two mesothelioma patients, with PD-L1 histoscore increasing from baseline levels of 17 and 1 to 47 and 23 after treatment, respectively (Fig. 9a). As both patients also showed a prominent infiltration of CD8+ T-cells to the tumor (Figs. 4 and 5) after treatment and a clear increase in the gene expression level of IFNγ in tumor (Fig. 9b), these findings suggest induction of dynamic adaptive changes in response to T-cell-derived IFNγ [44].

## Discussion

The primary endpoint of this study was to identify a safe and tolerated dose for phase II investigation. Since replication is essential to the mechanism of action of oncolytic adenoviruses, they typically have a non-linear toxicity curve. Consequently, no correlation was expected between dose and toxicity on the contrary to what would be expected in a study with a pharmacological agent. Therefore, no further dose escalation was performed even though no DLTs were observed in any dose cohort. This is in line with studies with other oncolytic viruses, where the MTD has not been reached [45–47]. It can be argued that MTD is not relevant based on the mechanism of action of oncolytic viruses where viral replication triggers the initiation of both direct cancer cell death and activation of the innate and adaptive immune system. The highest dose, 3 × 10^11^ VP/injection, was chosen as the recommended dose for further development, although any of the three doses examined in this study could potentially be used.

Cancer immunotherapeutic agents have been developed mainly as systemically administered drugs. However, most approaches, and especially the successful PD-1/PD-L1 blocking agents, are hampered by the commonly hypo-reactive local immune system in the tumor. Our strategy, in contrast, is to tackle the immune exhausted tumor microenvironment with locally delivered ONCOS-102 to prime anti-tumor immunity. By local administration, we maximise the safety of the treatment and circumvent the possibility of NAbs hampering efficacy. Indeed, some controversy is related to the significance of NAbs with regard to the efficacy of viral immunotherapy of cancer. While they clearly present a hurdle to systemic injections, leading to significantly reduced transfection of target cells, [48] the relevance of NAbs in a local setting is less clear. Importantly, we show that the efficacy of local treatment with ONCOS-102 is not hampered by the presence of pre-existing NAbs in the blood. Further, due to the marked liver-tropism of viruses, systemic administration requires very high doses of virus to saturate liver Kupffer cells and hepatocytes in order to achieve sufficient levels at the target tissues [49, 50]. Accumulation in the liver is accompanied by an acute phase immune response with elevated levels of cytokines such as IL-6 and TNF-α that can lead to life threatening systemic immunity [51]. We saw no indication of virus-related toxicity due to the elevated levels of IL-6 and IL-8, a safety aspect further strengthening the rationale behind our choice of local treatment instead of systemic.

The presence of tumor infiltrating lymphocytes (TILs), especially CD8+ T-cells, has been recognized as a marker of anti-tumor immune response across a wide range of cancers, [5, 24–27] and a positive correlation has been linked to high TIL counts at pre-treatment samples and good prognosis [28, 52, 53]. Further, pre-existing CD8+ T-cells seem to be required to achieve clinical efficacy with PD-(L)1 checkpoint blockade [7, 8, 54]. In established and progressing cancer, however, TILs often show an exhausted functional state similar to what is seen in chronic viral infection [55–58] due to persistent tumor-antigen load, adaptive responses in cancer cells and immunosuppressive factors in the tumor microenvironment. This is highlighted in last-line cancer patients where the immunosuppressive state of the tumor is often extensive, with exhausted (unfunctional) lymphocyte phenotype, high level of regulatory T cells or no indication of pre-treatment TILs [28, 52, 53, 59–62]. Thus, new complementary strategies are required to activate cellular antitumor immunity in immune cell-poor cancers.

This study demonstrates that ONCOS-102 is able to induce cellular antitumor immunity. We believe that this immune priming ability is a consequence of the innate immune system activation via pattern recognition receptors (PRRs) (e.g. toll-like receptors 2 and 9) and immunogenic cancer cell death that ONCOS-102 causes. Immunogenic cancer cell death is associated with the presentation of calreticulin on the cell surface and the release of natural adjuvants, specifically high-mobility group protein B1 (HMGB1) and ATP from within the dying cells, [9, 63] eventually leading to DC stimulation and subsequent activation of adaptive immune response [64]. We think that this “danger environment” created by ONCOS-102 [65] was essential in breaking the tolerance against tumor antigens that are essentially “self” and, by definition, either weakly immunogenic or functionally non-immunogenic due to the immunologically compromised tumor environment. Some encouraging previous data from local T-VEC treatment exist suggesting stimulation of both local and systemic tumor antigen-specific T cell immunity [20]. Unfortunately the small sample size and the absence of matching baseline samples remarkably limit the significance of these findings.

It is important to bear in mind that the small size (12 patients) and heterogeneity of the patient population in this phase I trial makes any definite conclusions regarding clinical benefit impossible. Yet, it is noteworthy that we show infiltration of CD8+ T-cells to tumors that were negative at baseline, and induction of systemic tumor antigen-specific T-cell response resulting from viral treatment. Importantly, the patients had late stage refractory diseases of varying origin with no further treatment options left to consider. A virus-induced cellular immune response in such a patient population is an encouraging proof of the ability of ONCOS-102 to awaken anti-tumor immunity in highly immune suppressed tumors. Further, this effect was seen in cancer types that differ from melanoma that is known to be highly immunogenic with high mutation rates that lead to frequent spontaneous immune cell infiltration to tumors and tumor-specific T cell responses [21, 66]. In case of viral immunotherapy, tumor antigens that are essentially “self” must compete against highly immunogenic viral antigens that are foreign, and this may limit the immune response to tumor, highlighting the importance of our findings [12]. Indeed, the choice of vector is critical as viruses differ in ways that affect their immunogenicity and oncolytic potential. Generally a highly immunogenic virus has a somewhat restricted oncolytic potential due to the brisk host immune responses, and vice versa [67]. Due to its immunogenic nature, ONCOS-102 is well suited for immunotherapy approaches.

Importantly, the two patients with the most prominent treatment-induced infiltration of CD8+ T-cells to tumor as well as a systemic anti-tumor cellular response had the best overall survival. Previously, some indication of favourable anti-tumor immunity was presented in a multicentre phase II study where metastatic melanoma patients were treated with T-VEC, an oncolytic herpes simplex virus coding for GM-CSF [20].

Treatment with ONCOS-102 also induced a T_H_1 type gene signature, and the presence of markers for T_H_1 polarization in the tumor has been implicated as important factor in predicting clinical outcome of patients [6, 36]. Interestingly, inflammatory cytokines, especially IFNγ, also cause dynamic adaptive changes of cancer cells [44, 68]. The immune inhibitory ligand PD-L1 on cancer cells is upregulated in response to T-cell-derived IFNγ, and engages PD-1 on T-cells, thereby attenuating their effector functions. We have shown that treatment with ONCOS-102 not only induces T-cell effector functions but also upregulates PD-L1 expression on tumors. This phenomenon is likely due to the CD8+ T-cells infiltrating the tumor as a response to the immunogenic cancer cell death caused by ONCOS-102. As biopsies represent a snapshot of the tumor immune microenvironment with temporal and dimensional restrictions, small samples may miss the relevant sites for PD-L1 expression and the time-point may be less than optimal for biologically relevant PD-L1 expression to occur [69]. To this end, it is noteworthy that this adaptive response could be seen in some patients after treatment, further highlighting the potential of ONCOS-102 to prime immune cell-poor tumors for PD-1/PD-L1 blockade.

## Conclusions

We show that locally administered ONCOS-102 is well tolerated and results in the induction of local and systemic CD8+ T-cell immunity against tumor in patients with treatment refractory and immune cell-poor cancers. Further, a dynamic adaptive change in cancer cells depicted in the form of PD-L1 up-regulation after treatment was seen, and together these results award further investigations into the combination of ONCOS-102 with other immune therapies such as PD-1/PD-L1 inhibitors.

## Methods

Patient characteristics, inclusion and exclusion criteria

Twelve patients with solid tumors refractory to available treatments were treated in this phase I clinical trial with ONCOS-102 (Table 1). Inclusion criteria included age over 18 years, a solid refractory tumor confirmed by histology and at least one tumor measurable by PET plus suitable for biopsy and WHO performance score of 0–1. Exclusion criteria included the use of high dose immune suppressive medication, vaccination with a live virus within 4 weeks of treatment, severe or unstable cardiac disease, known brain metastases, glioma, or central nervous system malignancy, HIV, history of hepatic dysfunction, cirrhosis or hepatitis, organ transplant or a clinically active infection or medical condition that, in the opinion of the principal investigator, might interfere with the investigation. The study was approved by the Hospital District of Helsinki and Uusimaa Ethics Committee, Department of Surgery. Written informed consent was obtained from patients prior to treatment, and the study was conducted in compliance with Good Clinical Practice guidelines.

### ONCOS-102

ONCOS-102 (previously called CGTG-102 and Ad5/3-D24-GMCSF) is a serotype 5 adenovirus that features a chimeric capsid with a serotybe 3 fiber knob for enhanced gene delivery to cancer cells and a 24 bp deletion in Rb binding site of E1A for cancer cell restricted replication. ONCOS-102 is armed with granulocyte-macrophage colony-stimulating factor (GM-CSF), a potent inducer of antitumor immunity. The construction of ONCOS-102 has been described elsewhere [13]. Manufacturing of ONCOS-102 for the trial was carried out in accordance with Good Manufacturing Practice (GMP).

### Study design and treatments with ONCOS-102 and cyclophosphamide

This was an exploratory, uncontrolled, non-randomised, un-blinded study of intratumoral (i.t.) and intravenous (i.v.) ONCOS-102, in conjunction with low dose cyclophosphamide (CPO), in patients with solid tumors that were refractory to available treatments (NCT01598129). The primary objective of the study was to determine the optimal dose of ONCOS-102 for use in Phase II and further development. As secondary objectives, the safety and tolerability of ONCOS-102 with low dose CPO were studied. Further, we sought to obtain preliminary evidence of disease control by ONCOS-102, as well as to determine the response rate to ONCOS-102 and the overall survival of patients treated in the trial. As exploratory endpoints, the effect of ONCOS-102 on immunological and biological correlates, especially cellular immune responses, were determined.

This was a classic 3 + 3 dose escalation study. The total i.t. doses of ONCOS-102 were 3 × 10^10^ virus particles (VP)/injection (low dose), 1 × 10^11^ VP/injection (mid dose), and 3 × 10^11^ VP/injection (high dose) on days 1, 4, 8, 15, 29, 57, 85, 113 and 141. I.t. injection encompassed intrapleural and intraperitoneal (i.p.) injection where appropriate. On Day 1 only, 20 % of the dose was administered intravenously (i.v.) and on subsequent days, 100 % was given i.t. I.t. injections were given by ultrasound guided needle. CPO 50 mg/day p.o. CPO (Baxter, Halle, Germany) was given daily until day 141, starting on day 2. PBMCs were collected before each ONCOS-102 administration and diagnostic positron emission tomography (PET) and computed tomography (CT) scans were performed on days 85 and 169.

### Tumor biopsies and blood samples

A core needle biopsy of the tumor was taken at baseline before the first treatment and one (day 29) and 2 months (day 57) after the treatment initiation. The sample was transferred immediately on a pre-cooled petri dish to sterile 0.9 % NaCl solution and divided into pieces for further analysis. For RNA-extraction, a part of the sample was immediately snap frozen on dry ice and stored in −80 °C. For immunohistochemistry (IHC) analysis, a part of the sample was placed in 10 % formalin buffer and kept at room temperature. Blood samples were collected from serum (3.5 ml in serum gel tube) and whole blood (2 ml in EDTA tube) before each treatment and 6 and 24 h after each treatment. Serum samples were centrifuged and frozen to -20 °C and whole blood samples were frozen to −20 °C after collection. Blood samples for leucocytes (32 ml before treatment initiation and 16 ml before each subsequent treatment in CPT tubes) were immediately processed for leucocyte isolation.

### Leucocyte isolation and IFNγ ELISPOT

The PBMCs were isolated using vacutainer cell preparation (Beckton Dickinson, Franklin Lakes, NJ, USA), and suspended into 1 ml CTL-Cryo^TM^C-reagent (Cellular Technology Limited, Shaker Heights, OH, USA). The cell concentration was counted in a haemocytometer-chamber (C-Chip disposable Haemocytometer, Digital Bio) under a 40 x objective. The live cells were separated from the dead cells by using Trypan blue solution (Amresco, Solon, OH, USA). CTL-Cryo^TM^A and CTL-Cryo^TM^B were combined and added to the cell solution according to the manufacturer’s instructions, and the cell solution was frozen on isopropanol container at −80 °C and further transferred to −140 °C.

Standard gamma IFN-ELISPOT was performed from pre- and post-treatment samples to analyse, whether the treatment induced tumor-specific cytotoxic T cell-responses. CD8+ T-cells purified with MACS® cell separation column (Miltenyi Biotech, Lund, Sweden) were pre-sensitized with peptide-pulsed, irradiated autologous PBMCs depleted of CD4+ and CD8+ T-cells. Pre-sensitized CD8+ T-cells were tested on day 10–12 by IFNγ ELISPOT assay for recognition of peptide-pulsed T2 cells or peptide-pulsed autologous antigen-presenting cells (EBV-transformed B cells or DCs). The number of cytokine-producing antigen-specific T-cells was evaluated using AID EliSpot Reader Classic ELR 07 (Autoimmun Diagnostika GmbH, Strassberg, Germany).

Based on the patient’s HLA haplotype, we analyzed CD8+ T-cell responses against NY-ESO-1, MAGE-A1, MAGE-A3 (MAGE-A1: (HLA-A1)EADPTGHSYp161-169 (HLA-A2)KVLEYVIKVp278-286, (HLA-B35)EADPTGHSYp161-169; MAGE-A3: (HLA-A1)EVDPIGHLYp168-176, (HLA-A2)FLWGPRALVp271-279, (HLA-A2)KVAELVHFL p112-120, (HLA-A35)EVDPIGHLYp168-176, (HLA-B18)MEVDPIGHLYp167-176 (HLA-B44)MEVDPIGHLYp167-176; NY-ESO-1: (HLA-A2)SLLMWITQCp157-165, (HLA-B35)MPFATPMEA p94-102, (HLA-B51)MPFATPMEAp94-102, (HLA-Cw3)LAMPFATPM p92-100) and/or known CD8 antigenic epitopes shown in the peptide database from van der Bruggen on http://www.cancerimmunity.org/peptide/. CD8+ T cell response against mesothelin for patient FI1-19 was analysed using overlapping long peptides kindly provided by Professor Markus Maeurer from Karolinska Institutet, Sweden.

### Immunohistochemistry

For analysis of different leucocyte populations on biopsy samples, three μm sections were cut from formalin-fixed and paraffin-embedded (FFPE) tissues and processed for immunohistochemistry performed with Ventana BenchMark XT immunostainer (Ventana Medical Systems, Tucson, AZ, USA). Rabbit monoclonal antibodies used were as follow: anti-CD8 clone SP57 (ready to use, Ventana), anti-CD4 clone SP35 (Cell Marque, Rocklin, CA, USA). Mouse monoclonal antibody for CD68 was clone KP1 (Dako, Glaustrup, Denmark), for CD11c clone 5D11 (Novocastra, Leica Microsystems GmbH, Wetzlar, Germany) and for CD19 clone LE-CD19 (Dako). Visualization was done using either UltraView Dabv3 (for CD8 and CD68), OptiView DAB IHCv3 (for CD4) or Envision K5007 (Dako) (for CD11c and CD19) with amplification (Ventana). The specimens were counterstained with hematoxylin and post counterstained with bluing reagent.

For quantitation, a color information based image processing methodology was applied. Samples were digitally scanned (3DHISTECH Ltd, Panoramic 250 FLASH, Budapest, Hungary and VCC-F52U25CL camera, CIS, Tokyo, Japan) and the images were compressed to a wavelet file format (Enhanced Compressed Wavelet, ECW, ER Mapper, Erdas Inc, Atlanta, Georgia) and archived online using Webmicroscope whole-slide image management platform (WebMicroscope, Fimmic Ltd, Helsinki, Finland) running with image server software (Erdas Apollo Image Web Server, Intergraph, Norcross, GA). Uneven background illumination was corrected, exclusively positively stained cellular regions were identified and possible unspecific staining was filtered out. Finally, the immunohistochemically stained samples were quantified by calculating a fraction of positively stained cellular region in the total tissue area. The image-processing pipeline was implemented in matrix laboratory (MATLAB, version R2012b) numerical computing environment.

For the analysis of PD-L1 molecule on tumor biopsy samples, four μm sections were cut from FFPE tissues, mounted on glass microscope slides and processed for immunohistochemistry using EnVision^TM^FLEX Target Retrieval System (Dako). Rabbit primary monoclonal PD-L1 XP® antibody (1:500) (clone EIL3N, Cell Signaling, Danvers, MA, USA) and FLEX + Rabbit (clone SM805, Dako) antibody as a secondary antibody were used and visualization was achieved by using a labelled polymer (FLEX/HRP) and DAB+ Substrate-Chromogen (Dako).

### Microarray analysis

Total RNA was extracted from snap-frozen core needle tumor biopsies taken at baseline and 1 and 2 months after the treatment initiation and gene expression profiling was performed by using HumanHT-12 Illumina microbead chips according to the standard protocols (Illumina Inc, San Diego, CA, USA). The probe-group intensity data were called using BeadStudio without background correction and normalization. The non-processed average signal values were then quantile-normalized, log2-transformed, and annotated using the package lumi (Bioconductor open source software). Chip-dependent batch-effects were removed using empirical Bayes methods [70]. Probes assigned to same Entrez gene identifier were averaged into a single expression estimate and probes left without gene information were removed. Differential expression analysis of the normalized data was performed using the Limma-package [71] by employing a paired *t*-test (pairing was done over samples originating from the same patient). The Storey’s Q-value adjustment [72] was used to correct data for multiple hypothesis testing.

### PET and CT scan

Changes in tumor metabolism and size was evaluated using fluorodeoxy-D-glucose (FDG) PET and diagnostic CT imaging at the screening visit, on Day 85 and at end of treatment (Day 169). The metabolic activity in PET was measured as maximum standardized uptake values (SUV max) from two measurement points in the tumor, or as total lesion glycolysis for patient FI1-14 for maximal reliability of the results. The response was evaluated using Response Evaluation Criteria in Solid Tumours version 1.1 (RECIST1.1) and PET response criteria.

### Adverse events

Physical assessment of the patients was done at every visit. Laboratory variables and other safety measures were analysed, i.e. standard CRP values, haematology abnormalities, liver enzymes ASAT and ALP, and blood potassium creatinine, sodium or INR after each treatment. Also blood pressure, pulse and SaO_2_ level were measured. Body temperature was measured and managed successfully with paracetamol or ibuprofen, if needed. Side effects were recorded according to common terminology criteria version 4 for adverse events (CTCAE ver. 4.0).

### Cytokines in blood

Cytokines were quantified from serum by using cytometric bead array system (CBA) (BD, Franklin Lakes, NJ, USA) for each cytokine. The analysis was performed according to the manufacturer’s instructions (BD Human Soluble Flex Set Kits), and the samples were run on BD LSRFortessa^TM^ flow cytometric cell analyser and the results were plotted using BD FACSDiva software.

### Quantitative real-time PCR (qRT-PCR)

DNA isolation for qRT-PCR was done using a Kit for isolation of DNA from body fluids (Generi Biotech, Hradec Kralove, Chzech Republic) according to the manufacturer’s protocol. A specific adenovirus PCR product was prepared from the genomic DNA of ONCOS-102 and cloned to a plasmid pCR4 TOPO TA (Invitrogen, Life Technologies, Thermo Fisher Scientific, Waltham, MA, USA) to be used as a positive control standard for the qRT-PCR assay. The qRT-PCR was done using primers Ad_dE1#1 CTATGCCAAAACCTTGTACCG and Ad_dE1#2 TCCTCACCCTCTTCATCCTC and probe Ad_E1#P TGATCGATCCACCCAGTGACGAC targeting the *E1* gene, specifically the region with the 24 bp deletion. A traditional lambda phage-target specific internal amplification control (primers PhgL_Q2#1 AAAAAGGATGAATCGCTTGGTGTA and PhgL_Q2#2 AATCCTGAATTTTCGGTGATG and probe PhgL_Q2#PCCATCGTGCCGCGACTGC) was used to avoid false-negative results [73].

### Titer of the neutralizing antibodies

The determination of neutralizing antibody titer from patient serum was done as described previously [74]. Briefly, a non-replicating luciferase-expressing Ad5/3lucI adenovirus was used to analyse the effect of neutralizing antibodies on the gene transfer efficacy of the virus, and thereby to evaluate the relative amount of neutralizing antibodies in patient serum before and after treatment. Ad5/3lucI was incubated with complement-inactivated patient serum dilutions (four-fold from 1:1 – 1:16384), and the mixture was added on A549 cells for luciferase activity measurement 24 h later. The Nab titer was determined as the lowest degree of dilution that blocked the luciferase gene transfer more than 50 % compared to virus alone.

### Statistical methods

Descriptive statistics for the safety and efficacy measures were calculated and displayed by dose group. Categorical data were presented by number of patients and percent for each category. Continuous data were presented by means and standard deviation (SD). Kaplan-Meier estimates were utilised for time-to-event analysis. AEs were coded using the Medical Dictionary for Regulatory Activities (MedDRA, version 15.0), and were summarised by system organ class (SOC) and preferred term (PT). Exploratory correlation analysis using Spearman’s method was applied to absolute values and fold-change from baseline in TILs vs. overall survival.

## Abbreviations

AE: adverse event
ALP: alkaline phosphatase
APC: antigen presenting cell
AST: aspartate aminotransferase
CPO: cyclophosphamide
CT: computed tomography or cancer-testis
CTCAE: common terminology criteria for adverse events
ELISPOT: enzyme linked immunospot assay
FDG: fluorodeoxy-D-glucose
FFPE: formalin-fixed and paraffin-embedded
GM-CSF: granulocyte macrophage colony stimulating factor
GMP: good manufacturing practice
HMGB1: high-mobility group protein B1
HSV1: herpes simplex virus 1
i.p.: intrapleural
i.t.: intratumoral
i.v.: intravenous
IFNγ: interferon gamma
IHC: immunohistochemistry
IL-10: interleukin-10
IL-6: interleukin-6
IL-8: interleukin-8
IRF1: interferon regulatory factor 1
NAb: neutralizing antibody
NK cell: natural killer cell
PBMC: peripheral blood mononuclear cell
PD-1: programmed death 1
PD-L1: programmed death 1 ligand
PET: positron emission tomography
PRR: pathogen-recognizing receptor
qRT-PCR: quantitative real-time PCR
RECIST1.1: response evaluation criteria in solid tumours version 1.1
SUV max: maximum standardized uptake value
TAM: tumor associated macrophage
TIL: tumor infiltrating lymphocyte
TNF-α: tumor necrosis factor alpha
VP: viral particle
